# Consensus Procedures in Oncological Imaging: The Case of Prostate Cancer

**DOI:** 10.3390/cancers11111788

**Published:** 2019-11-14

**Authors:** Stefano Fanti, Wim Oyen, Elisabetta Lalumera

**Affiliations:** 1 Department of Experimental, Diagnostic and Specialty Medicine—DIMES, University of Bologna, and Nuclear Medicine Division, Policlinico S.Orsola, 40138 Bologna, Italy; stefano.fanti@aosp.bo.it; 2 Department of Radiology and Nuclear Medicine, Rijnstate Hospital, 6815 Arnhem, The Netherlands; wjg.oyen@gmail.com; 3 Department of Biomedical Sciences, Humanitas University and Department of Nuclear Medicine, Humanitas Clinical and Research Center, 20126 Milan, Italy; 4 Psychology Department, Milano-Bicocca University, 20126 Milan, Italy

**Keywords:** nuclear medicine, guidelines, prostate cancer, overutilization, epistemology, consensus

## Abstract

Recently, there has been increasing interest in methodological aspects of advanced imaging, including the role of guidelines, recommendations, and experts’ consensus, the practice of self-referral, and the risk of diagnostic procedure overuse. In a recent Delphi study of the European Association for Nuclear Medicine (EANM), panelists were asked to give their opinion on 47 scientific questions about imaging in prostate cancer. Nine additional questions exploring the experts’ attitudes and opinions relating to the procedure of consensus building itself were also included. The purpose was to provide insights into the mechanism of recommendation choice and consensus building as seen from the experts’ point of view. Results: Regarding the factors likely to influence the willingness to refer a patient for imaging, the most voted were incorporation into guidelines and data from scientific literature, while personal experience and personal relationship were chosen by a small minority. Regarding the recommendations more relevant to prescribe an imaging procedure, it resulted the incorporation into guidelines promoted by scientific societies (59% of votes); these guidelines also resulted the more trusted. With respect to patients’ preferences considered when prescribing an imaging procedure, the most voted was accuracy, resulted more important than easy access and time to access to the procedure. The majority of the experts expressed the opinion that there is a scarce use of imaging procedures in prostate cancer. With respect to the most relevant factor to build consensus, it resulted the transparency of the process (52% of votes), followed by multidisciplinarity of contributors. The main obstacle to incorporation of modern imaging procedures into guidelines resulted the lack of primary literature on clinical impact. Conclusions: Firstly, the panelists portray themselves as having Evidence-Based Medicine oriented and scientifically inclined attitudes and preferences. Secondly, guidelines and recommendations from scientific societies, especially clinical ones, are positively taken into account as factors influencing decisions, but panelists tend to consider their own appraisal of the scientific literature as more relevant. Thirdly, in respect of overuse, panelists do not think that advanced diagnostic procedures are overutilized in the specific case of Prostate Cancer, but rather they are underutilized.

## 1. Introduction

Production of clinical practice guidelines (hereinafter “guidelines”) and consensus statements is of paramount importance for the medical community. This is because the availability of an increasing number of therapeutic and diagnostic options for many diseases requires careful choices, and evidence-based data such as meta-analyses are not always sufficient to drive appropriate use of medical options [1,2]. This is arguably more relevant in the field of advanced medical imaging, where the evaluation of a new diagnostic technique is often not obtainable from evidence cased on randomized controlled trials (RCT), for both conceptual and practical reasons [3].

Even though conspicuous efforts have been made recently to improve and standardize guidelines [4,5], they are still far from being a perfect tool, as philosophers of medicine and methodologists have outlined. They are inevitably outdated in respect of the development of new technologies, they are seldom followed in clinical practice, and they intrinsically need further clinical judgment in order to be appropriately applied to the individual patient’s case [6,7,8,9]. Moreover, it has recently been pointed out that scarce adherence to guidelines and recommendations favors self-referral and excessive compliance with patients’ requests, factors that may cause overuse of diagnostic procedures [10,11]. In addition to these problematic aspects, guidelines are likely to be affected by bias as they are promoted by professional organizations which may tend to select experts from among their members [12,13].

Literature on the functioning of guideline development groups and their attitudes (the view from inside) is relatively scarce [14,15,16]. Here we take Prostate Cancer (PC) as a case study and illustrate and discuss how experts themselves involved in a consensus conference judge the procedures of consensus building, referral, and compliance with guidelines.

Available diagnostic options at various stages of the disease require careful consideration. A number of imaging methods to study PC have been suggested. These include methods which have been available for decades, for example, computed tomography (CT), bone scintigraphy (BS), and trans rectal ultrasound (TRU), as well as methods which have been introduced more recently, such as whole-body magnetic resonance imaging (MRI), multiparametric MRI, and positron emission tomography (PET). However, there is significant disagreement about the usefulness of such approaches [17,18,19].

In light of this, the European Association for Nuclear Medicine (EANM), representing nuclear medicine specialists who have been at the forefront of advances in molecular imaging techniques for PC, launched EANM Focus 1 to develop consensus statements in PC. EANM Focus 1 was held in February 2018 and resulted in a consensus on molecular imaging for diagnosis and treatment of prostate cancer. The findings were published in Lancet Oncology in December 2018 [20].

The organizers of EANM focus 1 were also interested in knowing the experts’ attitudes and opinions relating to the procedure of consensus building itself as well as guidelines and their problematic aspects. For this reason, the final questionnaire included nine questions specifically focusing on these issues. This article reports and discusses the results of the aforementioned survey. Our goal is to provide insights into the mechanism of recommendation choice and consensus building as seen from the experts’ point of view. We are aware that experts’ declared attitudes and opinions may not be identical to the actual reasons motivating their decisions in practice. Nevertheless, we believe that their perception of their own role may be of interest in understanding the epistemology of consensus procedures, especially in the relatively new field of imaging epistemology. Moreover, we submit that experts’ reflection and self-appraisal on methodological and ethical issues may enhance their commitment to their scientific role.

## 2. Materials and Methods

EANM Focus 1 was based on a clinical questionnaire proposed and then agreed among the panelists. A modified Delphi process was used to generate consensus on the topics identified. Overall, the questionnaire contained 47 questions, and a 70% cut-off was adopted to determine consensus [21,22,23,24,25]. Three Delphi rounds were conducted, and consensus was achieved in 36 out of 47 clinical questions (77%), as detailed in [20].

In order to address the specific problem of bias from affiliation to a scientific society, a multidisciplinary expert panel was established (hereinafter “the panelists”). The panel consisted of 24 people with representation from all fields involved to ensure a similar number of oncologists (5), urologists (5), radiation oncologists (2), radiologists (4), and nuclear medicine specialists (7). All the panelists were selected on the basis of their international reputation. Furthermore, one pathologist, one molecular biologist, and one radio pharmacist were involved, as well as two patient advocates. The full list of panelists is enclosed in Appendix A.

During the first Delphi round, in addition to the 47 clinical questions, panelists were given nine additional questions specifically designed to evaluate the role of guidelines in respect of clinical practice and the overall approach to consensus building. Questions 48 to 55 are grouped by topic, and their results are shown below. An informal discussion of the findings, without statistical analysis, then follows.

## 3. Results and Discussion

### 3.1. Factors to Refer PC Patients for Imaging

Question number 48 was, “Which factors are likely to influence your willingness to refer a patient for an imaging procedure, assuming that availability is not limited?”, and panelists were asked to choose all that apply. Results are reported in Table 1.

Before moving to further consideration, it is necessary to clarify some possible implications deriving from this question (number 48), as well as from the next (number 49). Some of the answers, and in particular “personal relationship” and “personal experience”, may raise an ethical issue. If a “personal relationship” between a medical professional and the imaging professional has the ability to influence the outcome of the patient care, then we could have a major ethical violation. However, this was not the meaning of the answers and rather referred to professional trust that is developed when you personally know the imaging specialist and you are used to personally contacting him in case of any doubtful findings. Wording may seem somewhat vague, and therefore it is important to emphasize that all questions were reviewed and approved by all panelists, before being submitted, and no ethical concern was raised. The main reason to offer those possible answers was related to awareness that doctors may prescribe imaging exams not recommended by guidelines, for nonscientific reasons [26], and we were aimed at evaluating such possibility.

The panelists considered all the possible factors as potential influencers, but some of them were chosen by a small minority. In particular, the personal relationship with imaging professionals was regarded as a possible influencer by less than 20% of the panel. This indicates strong independence from a factor that may have either positive or negative implications.

Indeed, the personal relationship does not have much scientific value. It may be speculated that long-lasting collaboration may result in a fruitful professional relationship. Nonetheless, this factor seems less reliable than others. Surprisingly, the out of pocket cost of the examination for the patient gained the same low percentage of votes, while being a more relevant factor for appropriateness. It could be speculated that the experts regarded as minor the expense of an imaging procedure as compared with the overall costs of treatment. Further considerations regarding patient preference are detailed in question number 51 (see below).

A relatively low number of votes was also scored by the influence of the prescriber’s personal experience, confirming that all the factors relating to nonscientific data were regarded as less relevant. Indeed, the first three answers are clearly related to objective scientific facts and gained a clear preference among the experts, while the two answers relating to individual personal perception did not obtain many votes. Factors relating to patient perspective scored somewhere in between. These results are in line with a scientifically oriented approach of the panelists, who clearly preferred rational, evidence-based factors to less measurable issues.

Question number 49 was as question 48, but in this case, panelists were asked to choose one answer only: results are reported in Table 2.

When it comes to identifying the most relevant factor among the many proposed, there was a clear winner in data from scientific literature. Please note the sum of percentages is greater than 100% because several panelists, despite the question explicitly asking them not to, chose more than one answer. A formal consensus was reached regarding the most relevant factor (data from scientific literature), with almost 82% of the panelists voting for it. This consensus is in line with the principles of EBM (Evidence-based medicine) which consider scientific articles published in peer-reviewed journals as the most valuable source of evidence to decide whether to use a certain imaging procedure or not.

The panelists also considered incorporation into guidelines as a relevant factor, but to a minor extent. It should be mentioned that data from the literature is the prerequisite for proper incorporation into guidelines. Therefore, this factor implies the existence of the previous and most selected factor. The impression is that experts tend to prefer their own evaluation of original articles over guidelines, given that guidelines are only updated several months later.

There is much debate about the problems of timing required to have a new imaging method incorporated into PC guidelines. Previously, before the development of an EBM culture, conventional imaging methods such as CT and BS were incorporated without any formal evaluation of the positive effects on patient outcome. In the 1970’s, these methods were simply introduced and rapidly considered mandatory without a specifically designed clinical trial being undertaken. Thirty years later, rapid developments in the field of imaging still pose issues for guideline development. Indeed, new methods such as MRI and PET are available and have been adopted in clinical practice before evidence-generating clinical trials have been performed, resulting in a significant increase in their use. With the growth of medical imaging, the concern that not all examinations are necessary has increased, and it is argued that up to 40% of diagnostic imaging studies may be inappropriate^17-18^. Furthermore, as mentioned, there are intrinsic problems in building EBM literature for diagnostic imaging^11^ and this fact has led to significant disagreement in the field of PC imaging. At the Advanced Prostate Cancer Consensus Conference held in St. Gallen there was no consensus on most questions relating to advanced PC imaging [27]. The problem of reliability in the sources of recommendations was further investigated in the next question.

### 3.2. Source of Recommendations

Question number 50 was, “Which recommendations are more relevant for you when prescribing an imaging procedure?”: results are reported in Table 3.

There could be several reasons why a minority of the panelists chose the option of EBM/Health Technology Assessment-promoted guidelines. Firstly, in the area of prostate cancer, there is a complete lack of such guidelines, and the panel was surely aware of that even if the question did not focus on PC. A more general reason might be the resistance of clinicians to guidelines developed outside their professional control. See also question no. 54.

It is interesting to note that experts regard published studies as the most reliable evidence to influence their willingness to refer a patient for imaging (see question no. 49). On the other hand, EBM guidelines resulting directly from scientific data review are perceived as less reliable than scientific society guidelines.

It is noteworthy that not a single panelist considers local healthcare recommendations as relevant. Once again, this result could relate to the composition of the panel, which is made up of clinicians selected on the basis of their international reputation. This means they may not be keen to adhere to local rules that often tend to limit access to expensive and innovative imaging methods.

### 3.3. Patients’ Preferences

Question number 51 was “Which part of patients’ preference are you considering when prescribing an imaging procedure?”, and panelists were asked to choose all that apply: results are reported in Table 4.

The suggested answers to this question were slightly tricky because only the first four are genuinely related to patient preference and the fifth (accuracy of the procedure) relates to a scientific point of view. Indeed, it was this answer that received the largest majority of preferences (almost 82%), with the others being selected, but not to such a great extent. It is clear that this finding is related to the paternalistic approach of doctors to patients. When a final decision has to be taken, all patient preferences are duly taken into account, but the most relevant factor remains purely scientific evidence. In other words, the doctor is deciding in favor of the best diagnostic option, the value of which is more important than other factors.

### 3.4. Degree of Use of Imaging Procedures (See Below)

Question number 52 was, “Which is your overall opinion about the use of imaging procedures in PC?”: results are reported in Table 5.

Question 53 was, “Which is your overall opinion about the use of imaging procedures in PC for BCR/APC (Biochemical Recurrence/Advanced Prostate Cancer)?”: results are reported in Table 6.

### 3.5. Trustworthiness of Recommendations 

Question 54 was, “Which recommendations do you consider more trustworthy regarding imaging usage?”. Results are reported in Table 7.

This question is closely linked to question number 50, but with the emphasis placed on trustworthiness of recommendations. Once again there is a clear preference for guidelines promoted by clinical scientific societies. This point deserves further consideration. It seems that overall results are mainly influenced by the actual scenario of existing recommendations in the field. As already mentioned, guidelines promoted by clinical scientific societies are more common and more usual.

### 3.6. Building Consensus

Question number 55 was, “Which factor do you consider most relevant to build consensus on imaging procedures?”. Results are reported in Table 8.

The issue of consensus building is complex and controversial, with a number of factors involved and significant consequences for any healthcare system. Despite the fact that only one question in this questionnaire specifically covered the issue, some comments are necessary. The panelists clearly considered transparency of the process as the most relevant factor, thus favoring an EBM-oriented procedure. This is undoubtedly in line with current scientific opinion but contradicts the answers to question nos. 50 and 54, where EBM guidelines gained limited preferences. In other words, EBM methodology is widely recognized as the best option when making a recommendation, but there is resistance to guidelines promoted by EBM/HTA authorities. This fact is easily recognizable in the current practice of preparation of EAU guidelines, which are the most widely used in the treatment of PC.

### 3.7. Incorporation of Imaging into Guidelines

Question 56 was, “Which factor do you consider the most relevant obstacle for the lack of incorporation of modern imaging procedures into guidelines and clinical practice?”. Results are reported in Table 9.

This question is based on recognition that modern imaging procedures, such as MRI and PET, have gained widespread use in some countries despite a lack of incorporation into guidelines. Lack of primary literature on the clinical impact of modern imaging procedures is identified as the most relevant factor.

## 4. Conclusions

We cautiously assume that the panelists’ answers to the meta-questions show what they consider to be the right attitudes and preferences to have rather than the attitudes and preferences they actually have. The general picture that emerges is that experts, in their role as panelists, are adequately conscious of their role, that is, of which factors should be taken into account in deciding on imaging procedures within an EBM framework.

The results reported so far can be summarized as follows.

Firstly, the panelists show themselves to have EBM-oriented and scientifically inclined attitudes and preferences (questions 49, 55, and 56).

Secondly, guidelines and recommendations from scientific societies, especially clinical ones, are positively taken into account as factors influencing decisions, but panelists tend to consider their own appraisal of the scientific literature as more relevant and reliable. This is in line with a widespread need to rethink guidelines.

Thirdly, in relation to overutilization, panelists do not think that advanced diagnostic procedures are overutilized in the specific case of PC, but rather they are underutilized. Moreover, in line with the EBM-inclined attitude, they do not consider patient preferences as factors that should influence their decision whether to prescribe a test or not. This result suggests that the issue of overutilization of diagnostic procedures should be considered on a case-by-case basis. Furthermore, additional research should be done to compare the inclinations of medical specialists with those of general practitioners and patients with regard to this topic.

Fourthly, various problems specific to the field have also emerged. These include the relative scarcity of primary literature on the clinical effectiveness of some imaging tests and the predominance of the clinical importance of the procedure over its intrinsic diagnostic accuracy in evaluation and recommendation.

As a final and general suggestion, we submit that questions such as the ones we have posed and analyzed may, in general, enhance the experts’ self-appraisal of their role when actively participating in Delphi and consensus procedures, may act as a reminder of good scientific practices, and, therefore, may contribute to better performance on their part.

## Figures and Tables

**Table 1:** Consensus outcomes for question number 48.

Which Factors are Likely to Influence your Willingness to Refer a Patient for an Imaging Procedure, Assuming that Availability is not Limited?	
Incorporation into guidelines	76.2%
Data from scientific literature	90.5%
Study on cost-effectiveness	52.4%
Patients preferences	42.8%
Personal relationship with imaging professionals	19.0%
Personal experience	23.8%
Out of pocket cost to patient	19.0%
Abstain	4.3%
Unqualified to answer	4.3%

**Table 2:** Consensus outcomes for question number 49.

Which is the most Relevant Factor Influencing Referral to Imaging? Choose One	
Incorporation into guidelines	23.8%
Data from scientific literature	81.9%
Study on cost-effectiveness	9.5%
Patients preferences	4.7%
Personal relationship with imaging professionals	4.7%
Personal experience	0.0%
Out of pocket cost to patient	0.0%
Abstain	4.3%
Unqualified to answer	4.3%

**Table 3:** EANM (European Association for Nuclear Medicine) consensus outcomes for question number 50.

Which Recommendations are more Relevant for you when Prescribing an Imaging Procedure?	
Guidelines published by Evidence-Based Medicine/Health Technology Assessment authorities	13.6%
Guidelines promoted by scientific societies (imaging)	19.2%
Guidelines promoted by scientific societies (clinical)	59.0%
Local healthcare recommendation	0.0%
Proceeding of consensus multidisciplinary congresses	13.6%
None of the above	9.9%
Abstain	0.0%
Unqualified to answer	4.3%

**Table 4:** EANM consensus outcomes for question number 51.

Which Part of Patients’ Preference are you Considering when Prescribing an Imaging Procedure?	
Easy access to the procedure (distance and availability)	54.5%
Time to access to the procedure (waiting list)	45.4%
Cost of the procedure (irrespective whether or not it is covered by healthcare system or insurance)	22.7%
Out of pocket cost to patient	22.7%
Accuracy of the procedure (best available option)	81.8%
None of the above	9.9%
Abstain	0.0%
Unqualified to answer	4.3%

**Table 5:** EANM consensus outcomes for question number 52.

Which is your Overall Opinion about the Use of Imaging Procedures in PC?	
Overall there is excessive use of imaging procedures in PC	25.0%
There is excessive use of modern imaging procedures in PC	20.0%
Overall there is scarce use of imaging procedures in PC	10.0%
There is scarce use of modern imaging procedures in PC	60.0%
Use of imaging procedures in PC is optimal	10.0%
Abstain	8.7%
Unqualified to answer	4.3%

**Table 6:** EANM consensus outcomes for question number 53.

Which is your Overall Opinion about the Use of Imaging Procedures in PC for Biochemical Recurrence (BCR)/Advanced Prostate Cancer(APC)?	
Overall there is excessive use of imaging procedures in PC for BCR/APC	10.5%
There is excessive use of modern imaging procedures in PC for BCR/APC	15.8%
Overall there is scarce use of imaging procedures in PC for BCR/APC	5.2%
There is scarce use of modern imaging procedures in PC for BCR/APC	52.6%
Use of imaging procedures in PC is quite optimal for BCR/APC	15.8%
Abstain	9.0%
Unqualified to answer	4.5%

**Table 7:** EANM consensus outcomes for question number 54.

Which Recommendations do you Consider more Trustworthy Regarding Imaging Usage?	
Guidelines promoted by clinical scientific societies (European Association of Urology, or similar)	52.4%
Guidelines promoted by imaging scientific societies (European Association for Nuclear Medicine or similar)	19.2%
Guidelines promoted by EBM/HTA authorities	4.7%
Proceeding of consensus multidisciplinary congresses	14.3%
Equally all of the above	14.3%
Abstain	4.5%

**Table 8:** EANM consensus outcomes for question number 55.

Which Factor do you Consider most Relevant to Build Consensus on Imaging Procedures?	
Transparency of the process (evidence based medicine oriented)	52.4%
Participation of expert panelists (imaging specialist)	9.5%
Participation of multidisciplinary expert panelists (clinical specialist)	19.0%
Contribution of all stakeholders (specialists, patients, healthcare)	28.6%
Cost-effectiveness analysis	4.7%
Translational and primary literature data	9.5%
Abstain	0.0%
Unqualified to answer	4.5%

**Table 9:** EANM consensus outcomes for question number 56.

Which Factor do you Consider the most Relevant Obstacle for the Lack of Incorporation of Modern Imaging Procedures into Guidelines and Clinical Practice?	
Lack of primary literature on accuracy (Multicentric Prospective Trials)	9.5%
Lack of primary literature on clinical impact (Multicentric Prospective Trials)	66.6%
Lack of secondary literature (systematic reviews and meta-analyses)	4.7%
Lack of cost-effectiveness analyses	0.0%
Difficulties of access to modern imaging methods	19.0%
Difficulties in communications with imaging specialists	0.0%
Abstain	0.0%
Unqualified to answer	4.5%

## Appendix A—Expert Panel

**Name of Expert**	**Institute**	**Field of Expertise**
Gerald Antoch	University Hospital Düsseldorf, Germany	Radiology
Ian Banks	ECCO & European Men’s Health Forum	Patient Advocate
Alberto Briganti	San Raffaele University Hospital, Milan, Italy	Urology
Ignasi Carriò	Hospital de la Santa Creu i Sant Pau, Barcelona, Spain	Nuclear Medicine
Arturo Chiti	Instituto Clinico Humanitas, Milan, Italy	Nuclear Medicine
Noel Clarke	The Christie Hospital	Urological Oncology
Johann De Bono	The Institute of Cancer Research, London, United Kingdom	Medical Oncology
Matthias Eiber	Klinikum rechts der Isar, TUM, München, Germany	Nuclear Medicine
Karim Fizazi	Institut Gustave Roussy, Paris, France	Medical Oncology
Silke Gillessen	Kantonsspital St. Gallen, Switzerland	Medical Oncology
Sam Gledhill	Movember Foundation	Patient Advocate
Uwe Haberkorn	University Hospital Heidelberg, Germany	Nuclear Medicine
Ken Herrmann	Universitätsklinikum Essen, Germany	Nuclear Medicine
Rodney Hicks	Peter Mac Callum Cancer Institute, Melbourne, Australia	Nuclear Medicine
Frédéric Lecouvet	Clinique Universitaires Saint-Luc UCL, Brussels, Belgium	Radiology
Rodolfo Montironi	Ospedali Riuniti Ancona, Italy	Pathology
Joe O’Sullivan	Queen’s University Belfast, United Kingdom	Radiation Oncology
Piet Ost	Ghent University Hospital, Belgium	Radiation Oncology
Anwar Padhani	Mount Vernon Cancer Centre, London, United Kingdom	Radiology
Jack Schalken	Radboud UMC, Netherland	Molecular Biology
Howard Scher	Memorial Sloan Kettering Cancer Center, New York, USA	Medical Oncology
Bertrand Tombal	Cliniques Universitaires Saint-Luc; Belgium	Urology
Jeroen van Moorselaar	VU Medisch Centrum, Netherland	Urology
Hendrik Van Poppel	KU Leuven	Urology
Herbert Alberto Vargas	Memorial Sloan Kettering Cancer Center, New York, USA	Radiology
Jochen Walz	Institute Paoli-Calmettes, Marseille, France	Urology
Wolfgang Weber	Memorial Sloan Kettering Cancer Center, New York, USA	Nuclear Medicine
Hans-Jürgen Wester	Technische Universität München, Germany	Radiochemistry
